# Experimental Investigation on Cutting Characteristics and Surface Quality of TC18 Titanium Alloy in Longitudinal Ultrasonic-Vibration-Assisted Milling Under Dry Conditions

**DOI:** 10.3390/mi17070761

**Published:** 2026-06-23

**Authors:** Xiangyou Xue, Dongyan Shi, Biao Liu, Renjie Huang

**Affiliations:** 1 College of Mechanical and Electrical Engineering, Harbin Engineering University, Harbin 150001, China; xuexiangyou2581@hrbeu.edu.cn (X.X.); huangrenjie@hrbeu.edu.cn (R.H.); 2 Shenyang Aircraft Corporation, Shenyang 110850, China; liub014@avic.com

**Keywords:** TC18, ultrasonic vibration-assisted milling, dry cutting, cutting force, cutting temperature, surface roughness

## Abstract

This work presents a systematic investigation on dry milling of TC18 forged alloy using longitudinal ultrasonic vibration assistance. The effects of key parameters (cutting speed, feed per tooth, cutting depth and ultrasonic amplitude) on three-axis cutting forces, cutting temperature and surface quality are explored, and orthogonal experiments are conducted to determine the optimal parameter combination. Results reveal that increasing ultrasonic amplitude reduces cutting temperature by 31.8% and suppresses cutting forces effectively. Cutting depth and feed per tooth act as major influencing factors; the three-directional cutting forces drop by 31.1%, 56.7% and 22.9%, respectively. Surface roughness rises to 0.435 μm and 0.29 μm with growing feed per tooth and cutting depth, and decreases to 0.24 μm at higher cutting speeds. Under ultrasonic assistance, roughness increases slightly first and then declines remarkably. A threshold value exists for ultrasonic amplitude, and periodic tool–workpiece contact transforms strip textures into fish-scale morphologies. Proper parameter matching for ultrasonic milling lowers cutting forces and temperature, and improves surface quality of TC18 alloy. This study offers experimental data and theoretical references for relevant machining research.

## 1. Introduction

Titanium alloys possess comprehensive material advantages including low density, high specific strength, excellent high-temperature resistance and superior corrosion resistance, which have enabled their extensive applications in the manufacturing of aerospace military equipment, biomedicine and other high-end engineering fields [1,2,3,4]. Meanwhile, titanium alloys are characterized by a low deformation coefficient, high thermal strength and low elastic modulus, with their specific strength approximately twice that of alloy steels, further consolidating their application advantages in high-end manufacturing industries. Nevertheless, titanium alloys exhibit inherent machining challenges during mechanical cutting processes, such as high cutting temperature, severe springback deformation and strong chemical activity. These inherent drawbacks inevitably restrict the machining accuracy, processing efficiency and surface quality of titanium alloy components, rendering titanium alloys as typical difficult-to-cut materials in the mechanical machining field [5,6,7,8].

Compared with conventional machining processes, high-frequency and low-amplitude ultrasonic-assisted machining exhibits superior machining performance and application advantages in the cutting of advanced materials, which can significantly improve the machining quality of workpieces [9,10,11,12]. As a hybrid manufacturing technology that integrates ultrasonic special machining and conventional milling forming, ultrasonic vibration machining realizes efficient material removal and precision machining by applying high-frequency ultrasonic vibration to tools or workpieces [13,14]. Existing studies have demonstrated that the pulsed impact effect and intermittent cutting characteristics of ultrasonic machining can effectively restrain the excessive increase in cutting force and cutting temperature, optimize the surface integrity of workpieces, and refine the microstructure of the subsurface layer. Benefiting from the above technical advantages, ultrasonic-assisted machining has become a critical development direction and research hotspot in the field of high-efficiency and precision machining of difficult-to-cut materials such as titanium alloys [15,16].

At present, ultrasonic-vibration-assisted machining (UAM) of titanium alloys has been extensively investigated, with many findings validated in practical applications [17,18,19]. Sun et al. [20] proposed an approach to uncover the force-reduction mechanism in rotary ultrasonic-assisted side milling. By combining experiments and simulations, they systematically explored correlations among ultrasonic power, friction coefficient, and cutting force, providing theoretical support for precise force control. Focusing on Ti-6Al-4V, Pang et al. [21] evaluated the performance of longitudinal–torsional composite ultrasonic milling. Their results showed that its unique intermittent cutting characteristics reduced cutting forces by 46–86% compared to conventional milling (CM), while enhancing both workpiece quality and machining efficiency. Zhao et al. [22] examined how ultrasonic vibration direction affects TC4 machining performance, finding that axial vibration yields greater reductions in cutting force and better surface quality under controlled cutting speeds. For high-speed milling of TC4, Wang et al. [23] used orthogonal experiments to investigate parameter effects on surface roughness, clarifying each parameter’s influence characteristics and weights via range analysis. Niu et al. [24] studied longitudinal–torsional ultrasonic-assisted milling of Ti-6Al-4V, analyzing cutting force, temperature, and residual stress variations relative to CM. Orthogonal and single-factor tests confirmed that this method suppresses excessive increases in force and temperature, and optimizes surface residual compressive stress, offering a viable path for high-efficiency, precision machining of titanium alloys.

Existing studies have verified that ultrasonic-vibration-assisted milling can reduce cutting forces and cutting temperature, and improve surface quality during titanium alloy machining. Nevertheless, most relevant investigations have focused on TC4 titanium alloy [25,26,27,28]. As one of the high-strength titanium alloys widely adopted for aerospace structural components, TC18 possesses distinct cutting behaviors compared with conventional titanium alloys. To date, its machining responses under dry ultrasonic vibration cutting have not been thoroughly explored, and research targeting forged TC18 remains particularly limited. The few available reports on TC18 mainly discuss individual or a small set of cutting parameters, while comprehensive and systematic analyses are still lacking. Against this background, forged TC18 is selected as the workpiece material in the present work. Combining single-factor and orthogonal experiments under dry cutting conditions, this study systematically investigates the effects of cutting speed, feed per tooth, cutting depth and ultrasonic amplitude on cutting force, cutting temperature, surface roughness and surface topography. The underlying mechanisms responsible for the evolution of surface topography and roughness in ultrasonic-assisted cutting are also elaborated. This work is expected to provide theoretical references and technical guidance for the high-efficiency and precision machining of die-forged TC18 titanium alloy.

## 2. Materials and Methods

### 2.1. Characteristics of Longitudinal Ultrasonic-Vibration-Assisted Milling

**Figure 1:**
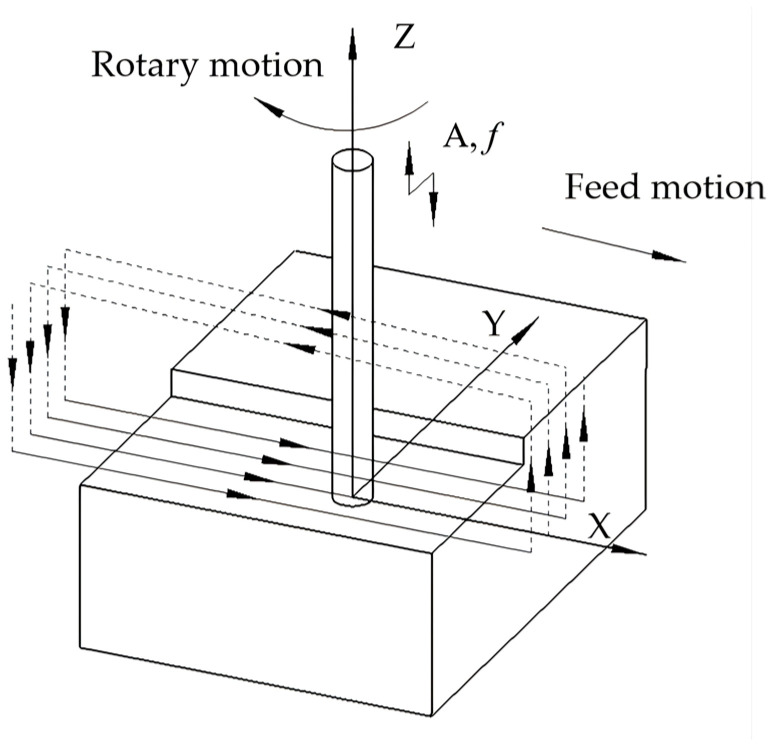
Principle of ultrasonic-assisted vibration milling.

In this work, longitudinal ultrasonic-vibration-assisted milling was employed for workpiece processing, which endows the cutting tool with high-frequency periodic reciprocating displacement along the axial direction. During machining, the axial vibration of the tool tip acts on the finished surface of the workpiece. The high-frequency vibration effect can effectively regulate the instantaneous cutting entry/exit velocity and undeformed chip thickness, thereby altering the cutting force characteristics and machined surface quality. The principle of longitudinal ultrasonic-vibration-assisted milling is shown in Figure 1. 

In the process of longitudinal ultrasonic-assisted milling, the motion trajectory of the tool cutting edge can be described by the following Equation (1) [29]:

(1) $$\left\{\begin{array}{l} x(t)=x_{0}+v_{w}t+R_{t}\sin[\frac{2n\pi }{60}+\frac{2\pi j}{N}]\\ y(t)=y_{0}R_{0}\sin[\frac{2n\pi }{60}+\frac{2\pi j}{N}]\\ z(t)=A\sin(2f\pi t) \end{array}\right.$$

**Figure 2:**
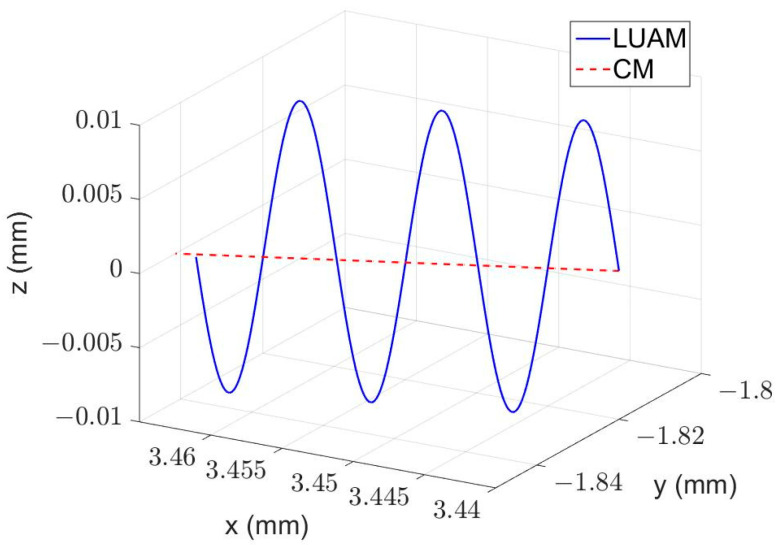
Longitudinal ultrasonic vibration tool tip motion trajectory (LUAM vs. CM).

In Equation (1), R_t_ is the tool radius, N denotes the number of cutting edges, j represents the j# cutting edge, *n* is the spindle speed, *f* refers to the vibration frequency, A is the axial vibration amplitude, *v_w_* is the feed speed, (*x*_0_, *y*_0_) corresponds to the initial position of the tool center. Compared with conventional milling (CM), the tool tip in axial ultrasonic-vibration-assisted milling performs additional high-frequency axial ultrasonic vibration on the basis of rotational primary motion and feed motion. The compound motion trajectory of a single cutting edge is presented in the corresponding Figure 2. All cutting edges simultaneously generate simple harmonic motion with a frequency of f and an amplitude of A along the circumferential direction.

### 2.2. Materials and Experimental Apparatus

**Figure 3:**
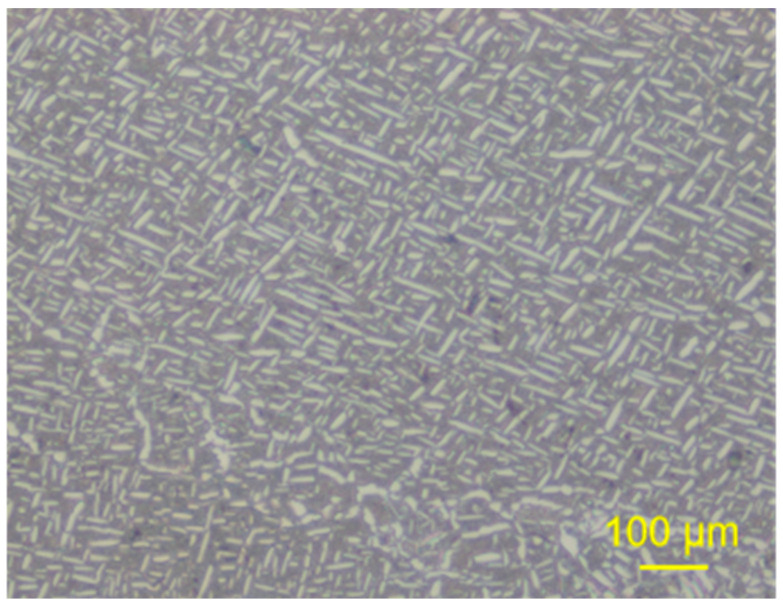
Uniform microstructure of the forging matrix.

In this study, forged TC18 titanium alloy (Guizhou Anda Aviation Forging Co., Ltd., Anshun, Guizhou, China) was selected as the experimental workpiece material. Its nominal chemical composition is Ti-5Al-5Mo-5V-1Cr-1Fe (mass fraction). As a typical near-critical α-β dual-phase titanium alloy, TC18 exhibits one of the highest strengths among titanium alloys under the annealed condition [30,31,32,33]. The detailed chemical composition is listed in Table 1. After forging, the forged blanks were inspected in strict accordance with industrial standards. Only the blanks with qualified performance indicators were adopted for subsequent experiments. The forged blank possesses a uniform and dense microstructure, and its micro-morphology is shown in the corresponding Figure 3.

**Figure 4:**
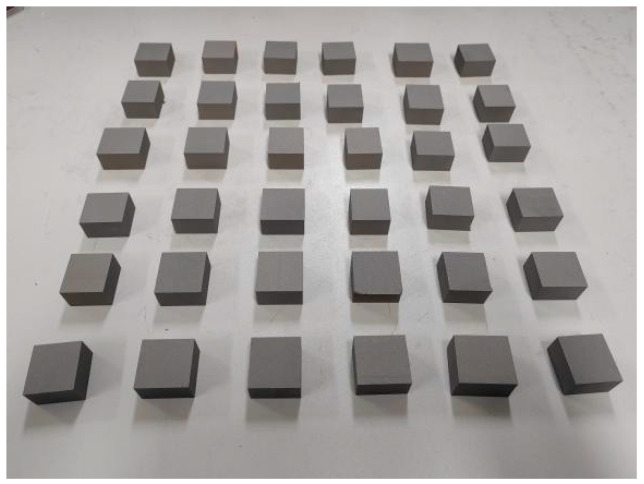
Wire-cut specimen.

To guarantee the reliability and accuracy of cutting force measurement and surface characterization, the qualified blanks were cut into cubic specimens with dimensions of 25 mm × 25 mm × 15 mm, as shown in Figure 4. To reduce experimental errors, all specimens were cut from identical regions of the raw blanks, so as to ensure the consistency of mechanical properties and minimize experimental deviations caused by metallurgical inhomogeneity. All cut specimens were subjected to vacuum stress-relief-annealing. The annealing temperature was set at 640 °C with a holding time of 1.5 h, followed by furnace slow cooling. This annealing procedure can effectively eliminate the residual stress induced by cutting processing and further improve the mechanical property uniformity of specimens [34]. The mechanical properties of TC18 titanium alloy are listed in Table 2.

**Figure 5:**
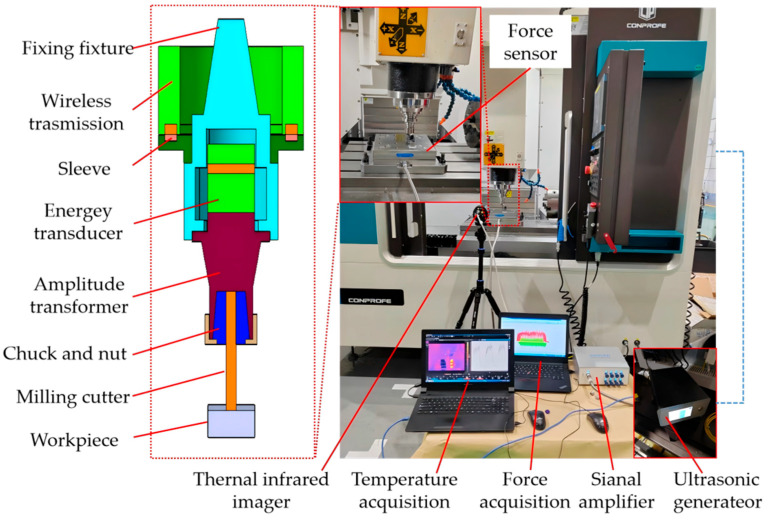
Ultrasonic vibration milling test setup.

All cutting experiments were conducted on a UGV-856 ultrasonic CNC machining center (Huizhuan Technology Group Co., Ltd., Dongguan, Guangdong, China), whose maximum spindle speed reached 10,000 rpm. The overall structure of the experimental setup is illustrated in the corresponding Figure 5. During the ultrasonic-assisted milling tests, the operating vibration frequency of the ultrasonic generator was steadily set to 20.4 kHz. The adopted ultrasonic vibration milling system is integrally composed of an ultrasonic generator, ultrasonic transducer, amplitude horn and cutting tool. In operation, the ultrasonic generator outputs high-frequency electrical signals, which are converted into mechanical energy via the ultrasonic transducer. Based on the inverse piezoelectric effect, high-frequency electrical signals are efficiently transformed into high-frequency mechanical vibrations. Subsequently, the mechanical vibration energy is transmitted through the amplitude horn. Owing to the structural differences in the horn, ultrasonic vibrations with customized parameters can be generated at the horn end and stably transmitted to the cutting tool.

In this work, an amplitude-adjustable ultrasonic vibration system was adopted. The longitudinal ultrasonic vibration generated by the system was transmitted to the tool tip through the ultrasonic tool holder, thereby realizing ultrasonic-assisted cutting. This ultrasonic-assisted module can be directly integrated and installed on conventional CNC milling machines and machining centers. Coil-inductive wireless energy transmission was applied between the ultrasonic power supply and the transducer. This design effectively avoids the limitation of non-rotatable power supply terminals and eliminates energy supply interference during the high-speed rotation of the transducer–horn assembly, ensuring the continuous and stable operation of the ultrasonic vibration system under spindle rotation conditions.

The cutting force measurement and data acquisition system (dynamometer 9255C, charge amplifier 5167A, Kistler Instruments AG, Winterthur, Switzerland) was rigidly mounted on the machine tool workbench. The prepared specimens were firmly clamped onto the force sensor via pressure plates to realize real-time collection and monitoring of milling force signals during cutting. The force sensor can accurately convert the mechanical pressure signals generated in the cutting process into electrical signals, which are subsequently transmitted to the signal amplifier for amplification. The operating frequency of the amplifier was set to three times the cutting vibration frequency to guarantee the accuracy and integrity of signal acquisition, and the measuring range of cutting force was defined as 450 N. The surface roughness of machined workpieces was measured using a roughness tester (Model MMD150HPG, Xi’an Wellson Precision Instrument Co., Ltd., Xi’an, Shaanxi, China), and the surface roughness was measured perpendicular to the surface texture direction, which corresponds to the X-axis direction in the coordinate system established in Figure 1. An infrared thermal imager (Model A700, FLIR Systems, Wilsonville, OR, USA) was adopted for cutting temperature measurement. This thermal imager is equipped with a 5-million-pixel resolution, a temperature measurement accuracy of ±2 °C, a spectral response range of 7.5~14 μm and an image sampling frame rate of 30 Hz, which satisfies the requirement for real-time capture of dynamic temperature variation during machining. To ensure the consistency and reliability of temperature testing, the thermal imager was fixed at a distance of approximately 1 m from the workpiece before formal tests, with its lens axis adjusted to be approximately horizontal to the cutting zone. During measurement, the ambient temperature was controlled at 25°C, and the surface emissivity of the workpiece was set to 0.34 [35]. Furthermore, the installation position of workpieces and the placement of the thermal imager remained unchanged throughout all cutting experiments. The surface morphology of the specimens was observed using a field-emission environmental scanning electron microscope (Quanta FEG 450, FEI Company, Hillsboro, OR, USA).

It should be noted that, although a certain deviation exists between the cutting temperature measured by the infrared thermal imager and the actual temperature in the cutting zone, the obtained results are still credible for evaluating the effects of different cutting parameters on temperature variation under a fixed emissivity setting.

A solid carbide end mill (Chengdu Great Wall Cutting Tool Co., Ltd., Chengdu, Sichuan, China) was adopted in this experiment. The detailed tool specifications are as follows: a diameter of 8 mm, a cutting edge length of 40 mm, an overall length of 78 mm, a corner radius of 1 mm, four cutting flutes, and an AlCrXN coating. After clamping, the tool overhang length was maintained at 30 mm.

### 2.3. Experimental Design

#### 2.3.1. Single-Factor Experimental Design

To investigate the influence laws of different cutting parameters and ultrasonic parameters on cutting force, cutting temperature and machined surface quality during ultrasonic vibration milling, ultrasonic vibration cutting experiments were conducted on forged TC18 titanium alloy in this study. Cutting speed, feed per tooth, cutting depth and ultrasonic amplitude were selected as experimental variables. Face milling was adopted in the experiments. Each group of tests was repeated no less than three times to reduce the measurement error of experimental results.

Prior to machining, each specimen was subjected to stress-relief-annealing to eliminate the interference of internal residual stress on experimental results and guarantee the consistency of experimental benchmarks. Meanwhile, the surface oxide layer of each specimen was removed by milling before experimental data recording. This operation avoided the uneven cutting thickness of the first cutting layer caused by the surface oxide layer and eliminated its adverse effects on experimental results.

To further improve the measurement accuracy, the average values of experimental results in the stable cutting region were calculated during data processing. The ranges of cutting parameters adopted in this experiment are listed as follows: cutting speed of 20~30 m/min, feed per tooth of 0.01~0.07 mm/z, cutting depth of 0.1~0.4 mm, and ultrasonic amplitude of 0~6 μm. The detailed cutting parameters are presented in Table 3.

#### 2.3.2. Orthogonal Experimental Design

In the multi-factor orthogonal cutting experiment, cutting speed, feed per tooth, cutting depth and ultrasonic amplitude were selected as four key experimental variables. Each factor was configured with four levels, and the specific parameter settings are summarized in Table 4.

**Figure 6:**
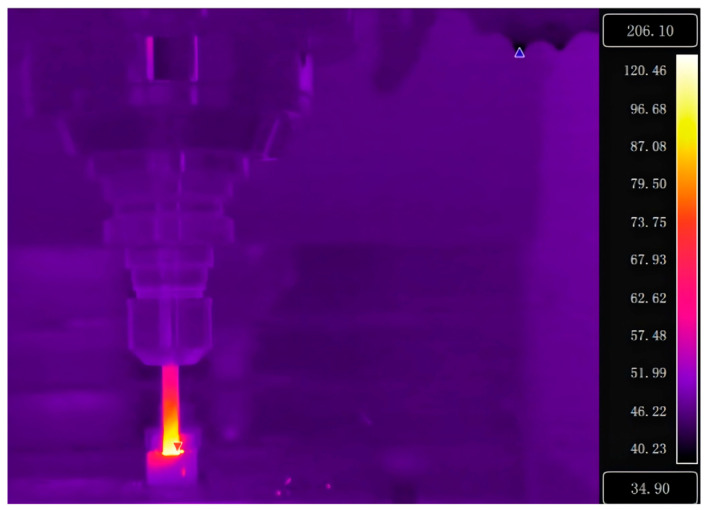
Infrared image for TC18 subjected to milling under dry conditions.

**Figure 7:**
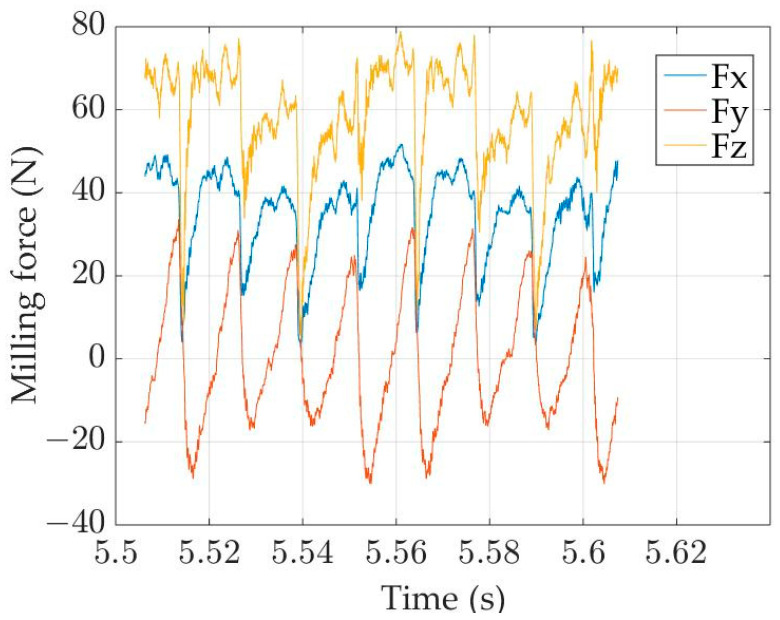
The dynamic variation trend of milling force.

During the cutting process, cutting force and cutting temperature were dynamically monitored and acquired in real time by testing devices, with the results presented in Figure 6 and Figure 7, respectively. The experimental results reveal that the cutting forces in all directions exhibit prominent high-frequency dynamic fluctuations. The figure presents the time-domain responses of three orthogonal cutting force components during the milling operation, acquired over a time window of 5.50~5.64 s. All three components exhibit periodic fluctuations synchronized with the cutting cycle of individual cutter flutes. Specifically, the axial cutting force (Fz) is the dominant component, fluctuating within the range of 60~80 N. The feed-direction force (Fx) is intermediate in magnitude, varying between 0 and 50 N, while the cross-feed force (Fy) shows the lowest amplitude, ranging from −30 to 30 N. The negative values observed in Fy are attributed to the coordinate system configuration in this up-milling setup, where the workpiece feed direction is opposite to the positive axis of the dynamometer. Each force peak corresponds to the engagement of a single cutting flute with the workpiece. Due to inherent variations in cutting conditions among the flutes, including instantaneous differences in undeformed chip thickness as the cutter rotates, the peak magnitudes generated by successive flutes are not identical. This behavior directly reflects the intermittent and cyclical nature of the milling process.

Fx and Fy demonstrate an alternating positive and negative variation tendency, So direct adoption of the average cutting force for data analysis would lead to the counteraction of positive and negative values, which inevitably deteriorates the test accuracy and analytical reliability. Accordingly, the root mean square of cutting force was adopted in this study to quantitatively characterize the cutting force magnitude, facilitating the comparative analysis of cutting force under diverse machining conditions. The sampling frequency of the infrared thermometer was set to 30 Hz, and the maximum temperature T_max_ within the cutting zone was selected as the critical indicator for the evaluation of cutting temperature.

To further explore the intrinsic correlation between cutting parameters and three key response indicators including cutting force, cutting temperature and surface roughness, a four-factor and four-level orthogonal experimental scheme based on the L16 (4^4^) was established in accordance with the fundamental principle of five-factor and four-level orthogonal design. A virtual blank column was introduced as the fifth factor to satisfy the standardized design requirements of orthogonal tests. A total of 16 independent experimental groups were arranged in this scheme. The detailed parameter configurations and corresponding measured results are summarized in Table 5.

## 3. Results

### 3.1. Single-Factor Analysis of Cutting Temperature

**Figure 8:**
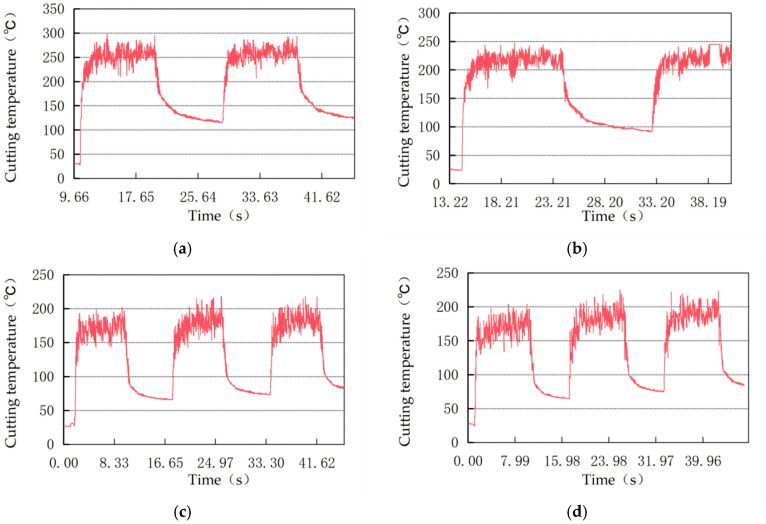
The variation pattern of the highest temperature during the cutting process with changes in ultrasonic amplitude (**a**) 0 µm, (**b**) 2 µm, (**c**) 4 µm, and (**d**) 6 µm.

To accurately characterize the temperature distribution during machining, a FLIR 700 thermal infrared imager (Model A700, FLIR Systems, Wilsonville, OR, USA) was utilized for cutting temperature measurement in this study. The infrared thermograms shown in Figure 6 are captured from the real-time infrared monitoring videos during the dry cutting process of TC18 titanium alloy. After calibrating the pixel range of heat source capture and setting the surface emissivity, the maximum temperature within the monitoring field of view could be extracted throughout the cutting process. It can be clearly observed from the infrared thermograms that the maximum cutting temperature is concentrated in the contact zone between the face milling cutter and the workpiece, which is highly consistent with the generation mechanism of cutting heat. Figure 8 presents the dynamic fluctuation curve of the maximum temperature in the cutting area acquired and output in real time by the temperature measurement software, which intuitively reflects the temporal variation law of cutting temperature during the entire machining process.

It can be analyzed from the cutting temperature fluctuation curve in Figure 8 that the cutting temperature rises sharply at the moment of each tool entry into the workpiece, and dynamically fluctuates within a specific range after entering the stable cutting stage. The underlying mechanism is explained as follows: intense interfacial friction occurs at the initial contact moment between the tool and workpiece. Meanwhile, the contact area is limited, and the heat generated by cutting cannot be rapidly conducted to the workpiece substrate, thereby resulting in a sharp instantaneous temperature increase. During the stable cutting process, the cutting edge maintains high-frequency intermittent contact and separation with the workpiece, which eventually leads to distinct temperature fluctuations and keeps the cutting temperature within a relatively stable interval.

Quantitative results extracted from Figure 8 demonstrate that the average cutting temperature is 249.4 °C when the ultrasonic amplitude is 0 μm. As the ultrasonic amplitude increases to 2 μm, 4 μm and 6 μm, the corresponding average cutting temperature decreases to 220.3 °C, 171.8 °C and 170.2 °C, respectively. This indicates that the cutting temperature decreases continuously with the increase in ultrasonic vibration amplitude. In terms of the intrinsic mechanism, ultrasonic vibration induces periodic contact–separation behavior between the tool and workpiece, which effectively optimizes the heat dissipation condition in the cutting zone and inhibits the accumulation of cutting heat, thereby realizing effective regulation of cutting temperature. Further comparison of temperature reduction amplitudes shows that the cutting temperature reduction reaches 31.8% when the ultrasonic amplitude increases from 0 μm to 6 μm. Nevertheless, the decreasing rate of cutting temperature is significantly weakened as the amplitude further increases from 4 μm to 6 μm. The above findings verify that with the continuous increase in ultrasonic amplitude, the cooling efficiency improves rapidly at the initial stage and then gradually levels off to a stable state.

**Figure 9:**
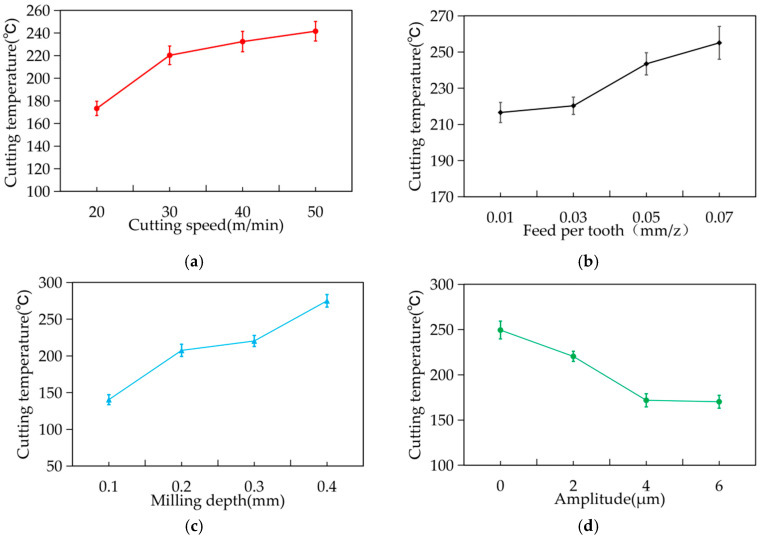
Effects of cutting parameters on cutting temperature. (**a**) Relationship between cutting temperature and cutting speed. (**b**) Relationship between cutting temperature and feed per tooth. (**c**) Relationship between cutting temperature and cutting depth. (**d**) Relationship between cutting temperature and ultrasonic amplitude. Error bars represent the standard deviation of three replicates.

To further investigate the influence mechanism and variation regularity of different cutting parameters on cutting temperature, the average values of the maximum cutting temperature under various levels of cutting speed, feed per tooth, cutting depth, and ultrasonic amplitude were extracted in this study. Based on these data, the variation curves of cutting temperature were plotted, as illustrated in Figure 9.

As depicted in Figure 9a, when the cutting speed increases from 20 m/min to 50 m/min, the cutting temperature rises from 173.3 °C to 241.6 °C, showing an overall significant upward trend, while the temperature rise rate gradually decelerates with the further increase in cutting speed. The underlying mechanism is that the increase in cutting speed substantially enhances the shear deformation rate of materials per unit time and the slip rate at the tool–chip interface, which further accelerates the conversion efficiency of deformation work and friction work into thermal energy, thereby leading to an elevation of cutting temperature. When the cutting speed reaches a certain critical threshold, the material softening effect can suppress the temperature rise rate to a certain extent, but it fails to alter the overall upward trend of cutting temperature.

It can be seen from Figure 9b,c that the cutting temperature exhibits an obvious monotonically increasing feature with the increase in feed per tooth and cutting depth. The main reason for the increase in cutting temperature induced by these two parameters is that the increase in both feed per tooth and cutting depth significantly increases the cutting force, and the thermal energy generated by the work performed by the cutting force increases substantially accordingly. Although the increase in cutting depth is conducive to the diffusion of heat from the cutting zone to the interior of the workpiece, the increase in heat flux density at the tool–chip interface still plays a dominant role. Meanwhile, the increase in feed per tooth or cutting depth tends to cause chip clogging, resulting in heat accumulation in the cutting zone and further exacerbating the rise in cutting temperature.

It can be observed from Figure 9d that the cutting temperature decreases significantly as the ultrasonic amplitude increases from 0 μm to 4 μm; however, when the amplitude continues to increase from 4 μm to 6 μm, the reduction amplitude of cutting temperature decreases obviously. The primary reason for this phenomenon is that ultrasonic vibration can induce periodic separation between the tool, workpiece, and chip, which effectively shortens the contact time among the three, thereby reducing the generation of frictional heat and deformation heat, and ultimately realizing the reduction in cutting temperature.

### 3.2. Single-Factor Analysis of Cutting Force

#### 3.2.1. Effect of Cutting Speed on Cutting Forces

**Figure 10:**
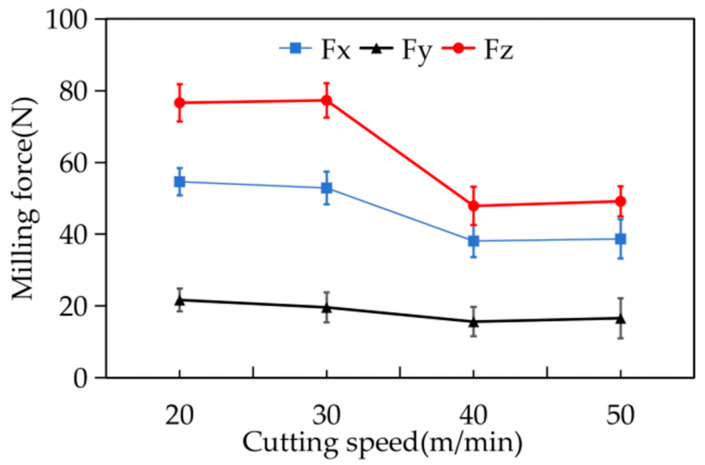
Effects of cutting speed variation on milling force (*f_z_* = 0.03 mm/z, *a_p_* = 0.3 mm, A = 2 μm). Error bars represent the standard deviation. Error bars represent the standard deviation of three replicates.

Under fixed processing parameters with constant feed per tooth, cutting depth and ultrasonic amplitude (cutting depth of 0.3 mm, ultrasonic amplitude of 2 μm, and cutting width of 2 mm), comparative cutting force experiments were conducted at different cutting speeds. The selected cutting speeds were set as 20, 30, 40 and 50 m/min, and the variation trends of cutting force with cutting speed are illustrated in Figure 10. The experimental results reveal that the cutting forces in three directions present an overall declining tendency with the increase in cutting speed. Within the range of 20~40 m/min, decrease from 55 N, 22 N and 77 N to 38 N, 15 N and 49 N, respectively. When the cutting speed further increases from 40 m/min to 50 m/min, the variation amplitude of cutting forces in all directions is greatly reduced, accompanied by a slight rebound. According to the attenuation analysis, when the cutting speed increases from 20 m/min to 40 m/min, the reduction rates of Fx, Fy and Fz reach 30%, 31% and 36%, respectively. Among them, Fz exhibits the most obvious decline, demonstrating that cutting speed exerts the dominant influence on Fz In the low-speed cutting range of 20~30 m/min, built-up edge is easily formed at the tool–chip interface, which changes the actual rake angle of the cutter, increases the cutting resistance, and maintains the cutting force at a relatively high level. A slight reduction in Fx and Fy occurs with a moderate increase in cutting speed, indicating an improved friction condition at the cutting interface. As the cutting speed rises to 30~40 m/min, the cutting temperature increases remarkably and the built-up edge gradually disappears. The alteration of the actual cutting angle contributes to a substantial reduction in triaxial cutting forces. In the high-speed range of 40~50 m/min, the continuous temperature rise intensifies the thermal softening effect of workpiece material and weakens the friction at the tool–chip interface, thereby enabling the cutting force to gradually stabilize.

#### 3.2.2. Effect of Feed per Tooth on Cutting Forces

**Figure 11:**
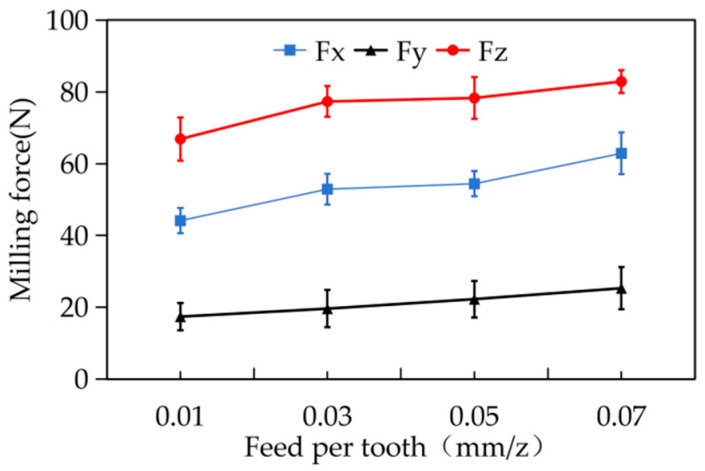
Effects of feed per tooth variation on milling force (*v*_w_ = 30 m/min, *a_p_* = 0.3 mm, A = 2 μm). Error bars represent the standard deviation.

Comparative cutting force tests under different feed per tooth were carried out with fixed cutting speed, cutting depth and ultrasonic amplitude. The constant machining parameters were set as follows: cutting speed of 30 m/min, cutting depth of 0.3 mm, ultrasonic amplitude of 2 μm and cutting width of 2 mm. The feed per tooth was designed as 0.01 mm/z, 0.03 mm/z, 0.05 mm/z and 0.07 mm/z, and the corresponding variation characteristics of triaxial cutting forces are presented in Figure 11. The experimental results indicate that the cutting forces in three orthogonal directions increase monotonically as the feed per tooth rises from 0.01 mm/z to 0.07 mm/z. Among the three components, the axial cutting force Fz maintains the maximum value, followed by Fx, while Fy remains the minimum throughout the tests. Quantitative analysis demonstrates that the growth rates of Fx, Fy, and Fz reach 42.47%, 45.45% and 23.95%, and the essential reason is that the increase in feed per tooth enlarges the radial cutting thickness, which further induces a remarkable rise in radial cutting resistance.

#### 3.2.3. Effect of Cutting Depth on Cutting Forces

**Figure 12:**
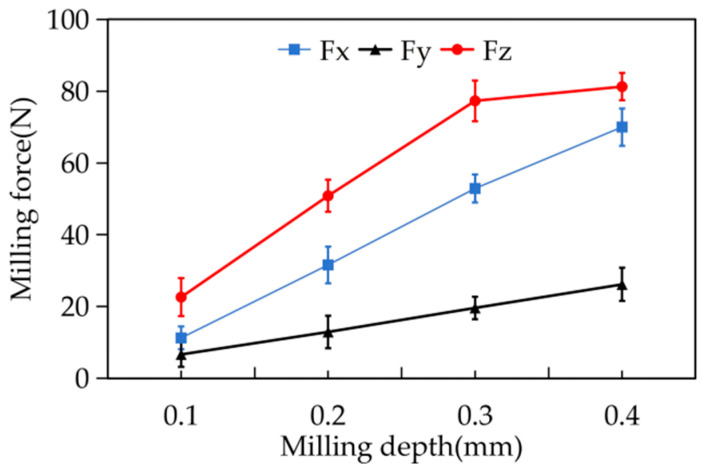
Effects of milling depth variation on milling force (*v*_w_ = 30 m/min, *f_z_* = 0.03 mm/z, A = 2 μm). Error bars represent the standard deviation.

With the cutting speed, feed per tooth, ultrasonic amplitude and other process parameters kept constant (cutting speed: 30 m/min, feed per tooth: 0.03 mm/z, ultrasonic amplitude: 2 μm, cutting width: 2 mm), the influence law of varying cutting depth on cutting forces was investigated. In this experiment, the cutting depth was set as 0.1 mm, 0.2 mm, 0.3 mm and 0.4 mm in sequence. The variation characteristics of three-directional cutting forces under different cutting depths are presented in Figure 12. The experimental results reveal that the three-directional cutting forces (Fx, Fy and Fz) increase monotonously as the cutting depth rises from 0.1 mm to 0.4 mm. Among them, the axial cutting force Fz maintains the highest value throughout the cutting process, while Fy remains the minimum. Meanwhile, Fz exhibits the largest variation range. This indicates that cutting depth exerts the most significant effect on the axial cutting force Fz, whereas its influence on the radial cutting force is relatively limited. When the cutting depth increases from 0.1 mm to 0.3 mm, all three-directional cutting forces rise sharply: Fx increases from 12 N to 53 N, Fy grows from 6.6 N to 19.6 N, and Fz rises from 22.6 N to 77.3 N. This phenomenon demonstrates that a slight increase in cutting depth within the shallow cutting depth range can remarkably change the contact state between the cutting tool and the workpiece, thereby leading to a dramatic rise in cutting resistance.

As the cutting depth further increases to 0.4 mm, the growth rate of cutting forces decreases obviously, and Fz gradually reaches a saturated state with only a slight increase from 77.3 N to 81.3 N. The underlying mechanism is that the continuous increase in cutting depth causes a notable temperature rise in the cutting zone, which induces an obvious thermal softening effect of workpiece material and ultimately weakens the growth magnitude of cutting forces.

#### 3.2.4. Effect of Ultrasonic Amplitude on Cutting Forces

**Figure 13:**
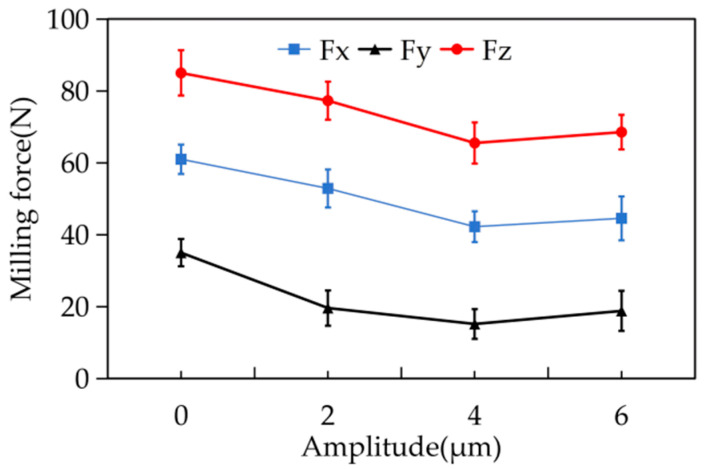
Effects of amplitude variation on milling force (*v*_w_ = 30 m/min, *f_z_* = 0.03 mm/z, *a_p_* = 0.3 mm). Error bars represent the standard deviation. Error bars represent the standard deviation of three replicates.

Under constant processing parameters, including a cutting speed of 30 m/min, feed per tooth of 0.03 mm/z, cutting depth of 0.3 mm and cutting width of 2 mm, the influence of ultrasonic amplitude on three-directional cutting forces was systematically investigated. Four ultrasonic amplitude levels were selected in this test, namely 0 μm, 2 μm, 4 μm and 6 μm. The variations in Fx, Fy and Fz under different ultrasonic amplitudes are illustrated in Figure 13.

As presented in Figure 13, the three components of cutting force all decrease evidently with the increase in ultrasonic amplitude, which is consistent with the fundamental mechanism of ultrasonic-vibration-assisted cutting. Essentially, the high-frequency vibration of the cutting tool enables periodic separation and contact between the cutting edge and workpiece. This periodic intermittent cutting behavior effectively reduces the average contact duration during material removal, resulting in a remarkable reduction in cutting forces.

Quantitatively, when the ultrasonic amplitude increases from 0 μm to 4 μm, Fx, Fy and Fz are reduced by 31.1%, 56.7% and 22.9%, and the cutting force is reduced to a certain extent. Nevertheless, a further increase in amplitude to 6 μm significantly slows down the decreasing rate of cutting forces, and a slight rebound of force values is even observed. This finding reveals that the separation effect induced by ultrasonic vibration is sufficiently saturated under high-amplitude cutting conditions. Consequently, further increasing the ultrasonic amplitude can hardly provide additional benefits for cutting force reduction, and the optimization effect gradually weakens.

### 3.3. Analysis of Orthogonal Experimental Results for Cutting Forces

#### 3.3.1. Range Analysis of Fx

**Figure 14:**
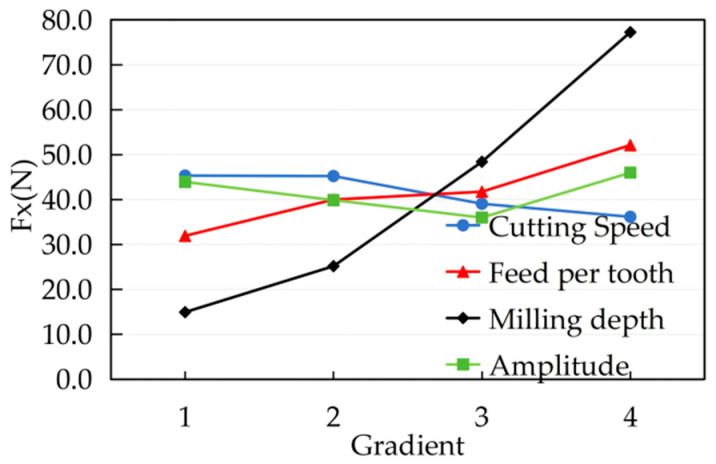
The trend of Fx under different horizontal parameters.

The range analysis results of the X-direction cutting force Fx are presented in Table 6, and its variation law is illustrated in Figure 14. The Fx value increases significantly with the rise in cutting depth and feed per tooth. In contrast, cutting speed and ultrasonic amplitude exert relatively weak effects on Fx. Under the investigated parameter combinations, the overall fluctuation of cutting force is slight, showing an overall variation trend of slight decrease at the initial stage followed by a slow increase.

According to the calculated range values of Fx, the results are obtained as follows: R(*a_p_*) = 62.342 > R(*f_z_*) = 20.189 > R(A) = 9.994 > R(*v_w_*) = 9.178. Accordingly, the primary and secondary order of the influence of each process parameter on Fx is determined as: *a_p_* > *f_z_* > A > *v_w_*. Combined with the above range analysis, the optimal process parameter combination is confirmed as A4B1C1D3 (*v_w_* = 50 m/min, *f_z_* = 0.01 mm/z, *a_p_* = 0.1 mm, A = 4 μm). Verified by cutting experiments, the X-direction cutting force Fx under this optimal parameter combination is only 9.8 N, which is remarkably lower than all test data in the orthogonal experimental groups. This result effectively verifies the rationality and feasibility of the optimized parameter combination.

#### 3.3.2. Range Analysis of Fy

**Figure 15:**
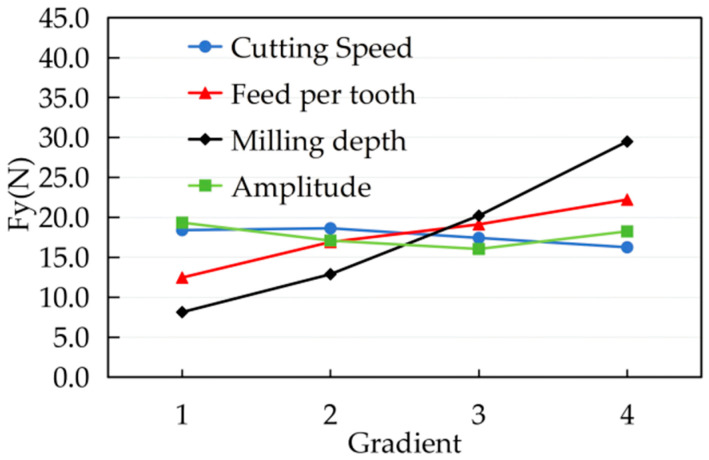
The trend of Fy under different horizontal parameters.

The range analysis results of the Y-direction cutting force are listed in Table 7, and the corresponding variation trends are shown in Figure 15. The value of Fy evidently increases with the increase in cutting depth and feed per tooth. Cutting speed and ultrasonic amplitude present limited disturbance effects on Fy, resulting in a small overall fluctuation range. Specifically, Fy decreases gradually as the cutting speed increases, while it first declines and then rises with the increase in ultrasonic amplitude.

The range calculation values of Fy are summarized as: R(a_p_) = 21.367 > R(*f_z_*) = 9.765 > R(A) = 3.3 > R(*v_w_*) = 2.381. On this basis, the influencing priority of each process parameter on the Y-direction cutting force Fy is determined as *a_p_* > *f_z_* > A > *v_w_*. Combined with the above analytical results, the optimal parameter combination is selected as A4B1C1D3 (*v_w_* = 50 m/min, *f_z_* = 0.01 mm/z, *a_p_* = 0.1 mm, A = 4 μm). The verification cutting experiments demonstrate that the Y-direction cutting force Fy under this optimal parameter set is only 5.1 N, which is significantly lower than all experimental data in the orthogonal tests. This sufficiently confirms the effectiveness and rationality of the optimized parameter combination.

#### 3.3.3. Range Analysis of Fz

**Figure 16:**
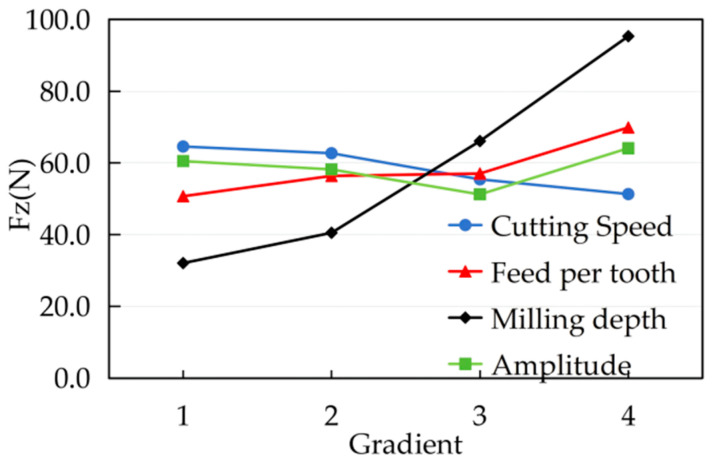
The trend of Fz under different horizontal parameters.

The range analysis results of the Z-direction cutting force Fz are presented in Table 8, and its variation characteristics are illustrated in Figure 16. The Fz value increases evidently with the rising of cutting depth and feed per tooth. As the cutting speed increases, Fznshows an overall decreasing tendency, while it exhibits a variation trend of first decreasing and then increasing with the growth of ultrasonic amplitude.

The range calculation results of Fz are as follows: R(*a_p_*) = 63.262 > R(*f_z_*) = 19.167 > R(*v_w_*) = 13.262 > R(A) = 12.885. Accordingly, the influencing sequence of various process parameters on Fz is determined as *a_p_* > *f_z_* > *v_w_* > A. Based on the above comprehensive analysis, the optimal process parameter combination is ultimately selected as A4B1C1D3 (*v_w_* = 50 m/min, *f*_z_ = 0.01 mm/z, *a_p_* = 0.1 mm, A = 4 μm). The verification results of cutting experiments indicate that the Z-direction cutting force Fz under this optimal parameter combination is only 26.8 N. This value is distinctly lower than all measured data in the orthogonal tests, which further fully validates the reliability and optimization rationality of the selected parameter combination.

#### 3.3.4. Variance and Range Analysis of Cutting Force

To examine the significance of cutting parameters on the cutting forces in different directions, an analysis of variance (ANOVA) was performed on the measured forces in each axis, with the results presented in the corresponding tables. For this orthogonal test design, each factor has three degrees of freedom (DoF = 3), and the analysis was conducted at a 95% confidence level. According to the F-test criterion, the critical value of the F-distribution is F_0.05_(3,3) = 9.28. As shown in Table 9, cutting depth exerts a significant effect on the cutting force in the X-direction. From Table 10, both feed per tooth and cutting depth significantly influence the Y-direction cutting force. Meanwhile, Table 11 indicates that only cutting depth has a statistically significant impact on the Z-direction cutting force. Combining the results from Table 9, Table 10 and Table 11, it is evident that feed per tooth and cutting depth are the dominant factors affecting the cutting forces. This finding also confirms that the magnitude of the cutting force is fundamentally governed by the material removal rate, which is directly determined by feed per tooth and cutting depth.

### 3.4. Single-Factor Experimental Analysis of Surface Roughness

**Figure 17:**
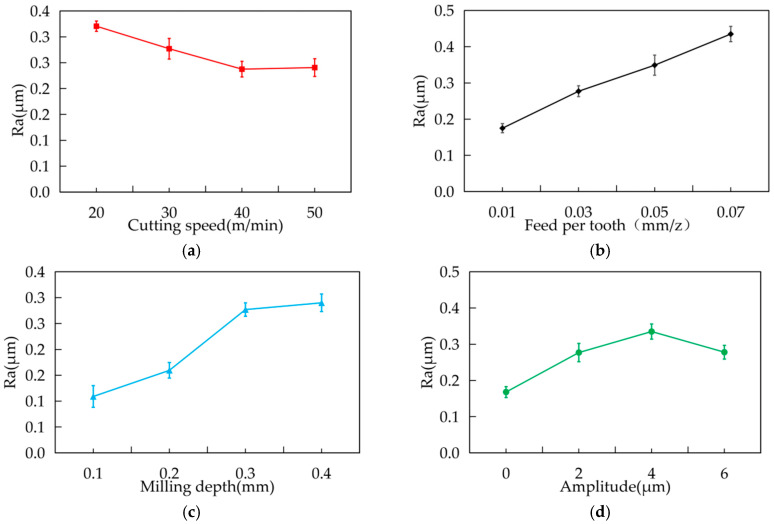
Effects of cutting parameters on surface roughness (**a**) Relationship between surface roughness and cutting speed; (**b**) relationship between surface roughness and feed per tooth; (**c**) relationship between surface roughness and cutting depth; (**d**) relationship between surface roughness and ultrasonic amplitude. Error bars represent the standard deviation.

The influence laws of ultrasonic vibration milling parameters on the surface roughness of workpiece are presented in Figure 17. The surface roughness reaches 0.33 μm at a cutting speed of 20 m/min. When the cutting speed increases from 20 m/min to 40 m/min, the surface roughness gradually decreases from 0.33 μm to 0.23 μm. With a further increase in cutting speed, the surface roughness slightly rebounds to 0.24 μm, exhibiting a marginal increasing tendency.

This variation indicates that increasing cutting speed within a reasonable range can effectively improve workpiece surface quality and reduce surface roughness. The underlying mechanism can be elaborated as follows. Under high-speed cutting conditions, the plastic deformation of materials is remarkably weakened. The elevated cutting temperature softens the surface material and mitigates interfacial friction. Meanwhile, a higher cutting speed can restrain the formation and adhesion of built-up edges, thus acquiring a flatter machined surface. Nevertheless, excessive increase in cutting speed will exacerbate the vibration of cutting systems and intensify the impact and friction between the flank face of milling cutter and machined surface, which eventually deteriorates the surface machining quality of workpieces.

As the feed per tooth increases from 0.01 mm/z to 0.07 mm/z, the surface roughness rises monotonically from 0.175 μm to 0.435 μm, demonstrating that feed per tooth exerts a significant positive effect on surface roughness. Specifically, the increase in feed per tooth raises the theoretical residual height of tool cutting trajectories, and simultaneously induces the growth of instantaneous cutting force and the aggravation of cutting force fluctuation. These behaviors generate deeper cutting marks and vibration textures on the machined surface, leading to a prominent increase in surface roughness.

With the cutting depth increasing from 0.1 mm to 0.4 mm, the surface roughness gradually rises from 0.1 μm to 0.28 μm and then tends to stabilize. The increment of cutting depth substantially increases the overall cutting load and induces severe cutting vibration, which further impairs the surface integrity of workpiece and results in a continuous increase and subsequent stabilization of surface roughness.

In addition, during the increase in ultrasonic amplitude from 0 μm to 6 μm, the surface roughness follows an overall trend of initial increase and subsequent decrease. At low amplitude levels, ultrasonic excitation tends to trigger micro-fluctuations of cutting force and cause non-uniform material removal, thereby degrading the surface machining performance. As the ultrasonic amplitude further increases, a distinct periodic intermittent separation effect occurs between tool and workpiece, which efficiently weakens the continuous friction between the tool flank and workpiece surface. Moreover, high-amplitude ultrasonic vibration delivers a high-frequency ironing and finishing effect on the machined surface layer, inhibiting surface micro-defects. Consequently, the surface roughness is reduced, and the surface quality of workpiece is effectively optimized.

### 3.5. Surface Roughness Analysis of Orthogonal Test Results

The results of the analysis of variance (ANOVA) for the surface roughness Ra of machined workpieces are presented in the Table 12.

For this four-factor, four-level orthogonal design, each factor has three degrees of freedom. With a 95% confidence level, the critical value of the F-distribution is F_0.05_(3,3) = 9.28. Based on the ANOVA results, within the selected range of cutting parameters, cutting speed and cutting depth exert statistically significant effects on surface roughness, while feed rate and ultrasonic amplitude show weaker influences. To further clarify the individual contributions of each cutting parameter to surface roughness, a range analysis was conducted.

**Figure 18:**
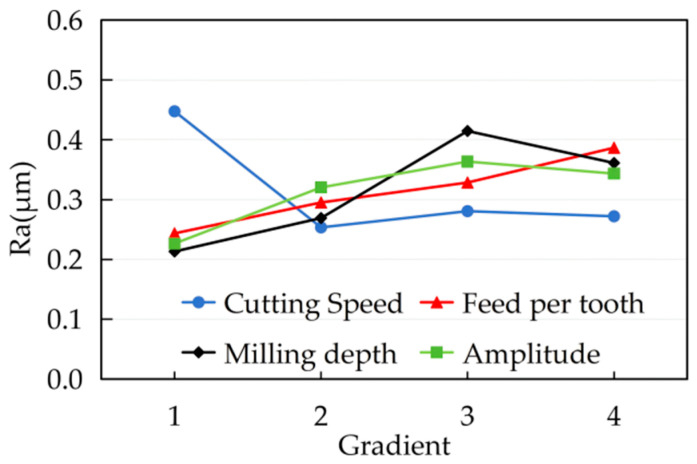
The trend of Ra under different horizontal parameters.

The range analysis results of surface roughness Ra are listed in Table 13, and its corresponding variation tendency is presented in Figure 18. With the elevation of cutting speed, the workpiece surface roughness generally decreases first and then tends to be stable, revealing that an appropriate increase in cutting speed within a certain range can effectively improve the machined surface quality. Increases in cutting depth and feed per tooth both lead to an overall upward trend of surface roughness. Nevertheless, when the cutting depth exceeds the critical value, a further increase in cutting depth contributes to a slight reduction in surface roughness. The intrinsic mechanism is that a sufficiently large cutting depth induces a prominent temperature rise in the cutting zone, which enhances the thermal softening effect of workpiece materials. This effect reduces the cutting force, frictional resistance and cutting vibration intensity, thereby lowering the final surface roughness.

In addition, the surface roughness exhibits a variation characteristic of initial increase followed by a gradual decline with the rising ultrasonic amplitude. This phenomenon is primarily attributed to the ironing and strengthening effect induced by high-amplitude ultrasonic vibration on the machined surface, which can effectively optimize surface morphology and improve the surface integrity of the workpiece.

According to the range analysis of surface roughness, the range values satisfy R(*a_p_*) = 0.201 > R(*v*_w_) = 0.176 > R(*f*_z_) = 0.143 > R(A) = 0.137. Accordingly, the influencing significance of each factor on surface roughness is ranked as follows: *a_p_* > *v_w_* > *f_z_* > A. Based on the above analytical results, the optimal parameter combination for the present machining condition is determined as A2B1C1D1, corresponding to a cutting speed *v*_w_ of 30 m/min, a feed per tooth *f*_z_ of 0.01 mm/z, a cutting depth a_p_ of 0.1 mm, and an ultrasonic amplitude A of 0 μm. The cutting experimental verification results show that the surface roughness under this optimal parameter combination is Ra 1.44 μm, which is significantly lower than all measured data in the orthogonal test. This further fully verifies the reliability of the selected parameter combination and the rationality of the optimization.

### 3.6. Analysis of Surface Morphology

**Figure 19:**
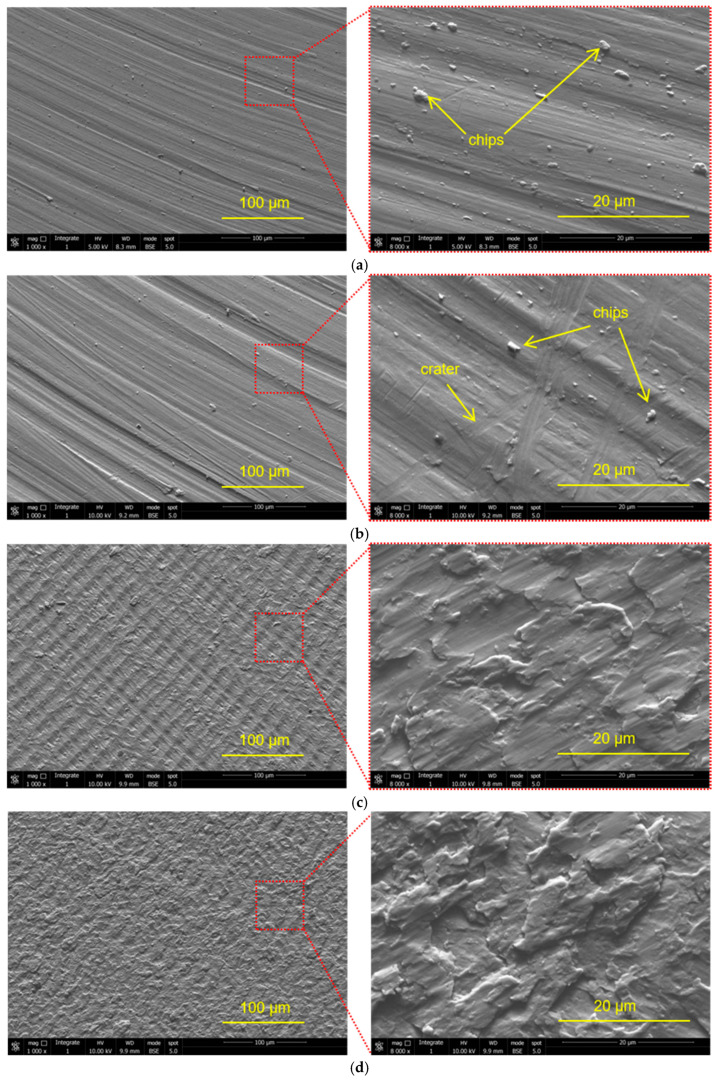
Surface topography after longitudinal ultrasonic vibration milling. (**a**) A = 0 μm; (**b**) A = 2 μm; (**c**) A = 4 μm; (**d**) A = 6 μm.

To explore the influence of ultrasonic amplitude on surface topography in face milling, face cutting tests were implemented at ultrasonic amplitudes of 0, 2, 4 and 6 μm while keeping other cutting parameters constant. The cutting speed was set at 30 m/min, the feed per tooth was 0.03 mm/z, the cutting depth was 0.3 mm, and the cutting width was 2 mm. The surface features of machined specimens were characterized via scanning electron microscopy (SEM), and the corresponding results are presented in Figure 19.

It can be seen that ultrasonic amplitude exerts a distinct effect on the machined surface morphology. At the amplitude of 0 μm, namely under conventional cutting conditions, prominent feed marks in the form of continuous band-like textures can be found on the workpiece surface, along with a certain amount of tiny adhered chips. When the amplitude rises to 2 μm, the overall surface state is similar to that obtained by conventional cutting. Deeper band-like marks and a small quantity of residual chips are still observable, and shallow cross-distributed depressions also emerge. Such depressions are presumably generated by the extrusion deformation induced by ultrasonic vibration. As the amplitude further increases to 4 μm, regular grid-like tool path textures form along the original band-like feed marks, while the banded feed traces remain visible. When the amplitude reaches 6 μm, the surface morphology changes dramatically. Impact and ironing traces caused by the cutting edge appear on the machined surface, and the surface is covered with fish-scale micro-textures. In this case, the original band-like feed marks are barely distinguishable, and no obvious chip adhesion can be detected and the surface quality of the workpiece is effectively improved.

From the above observations and analysis, it can be concluded that the cutting process in conventional machining is continuous, resulting in continuous band-like textures on the machined surface. With increasing ultrasonic amplitude, irregular craters initially appear on the surface. When the amplitude reaches 6 μm, the periodic tool entry–exit behavior is intensified, the band-like textures disappear, and regular fish-scale patterns are formed on the surface. The boundaries of these fish-scale units correspond to the traces left at the moments when the tool separates from the workpiece.

## 4. Conclusions

This work experimentally investigated the longitudinal ultrasonic-vibration-assisted milling (LUAM) of TC18 die-forged alloy under dry cutting conditions, with a focus on the evolution of cutting forces, cutting temperature, and machined surface quality. Combining single-factor and orthogonal experimental designs, the effects of key process parameters, i.e., cutting speed, feed per tooth, cutting depth, and ultrasonic amplitude, on three-dimensional cutting forces, cutting temperature, and surface roughness were systematically analyzed. The main conclusions are summarized as follows:

1. The experimental results revealed that cutting temperature gradually decreases with increasing ultrasonic amplitude. Specifically, when the amplitude increased from 0 μm to 6 μm, the maximum reduction in cutting temperature reached 31.8%. This phenomenon is primarily attributed to the periodic contact and separation between the cutting edge and the workpiece surface, which effectively enlarges the heat dissipation space and improves the cooling efficiency in the cutting zone.
2. In LUAM, the variation in cutting forces exhibited strong parameter dependence. Cutting forces decreased significantly with increasing cutting speed and ultrasonic amplitude, whereas they increased with higher feed per tooth and cutting depth. When the ultrasonic amplitude was increased from 0 μm to 4 μm, the three-directional cutting forces (Fx, Fy, and Fz) were reduced by 31.1%, 56.7%, and 22.9%, respectively. This reduction is mainly due to the periodic separation between the cutting edge and the workpiece, which shortens the effective contact time and consequently lowers the average cutting force.
3. The evolution of surface roughness was closely correlated with process parameters. As the cutting speed increased, surface roughness gradually decreased, while higher feed per tooth and cutting depth led to a monotonic increase in roughness. When the ultrasonic amplitude increased from 0 μm to 6 μm, surface roughness first increased and then decreased, indicating the existence of a critical amplitude for the effect on surface roughness.
4. With the introduction and increase in ultrasonic amplitude, the surface morphology evolved from continuous band-like milling textures to grid-like and fish-scale micro-textures. Meanwhile, the chip-breaking performance was enhanced, leading to an overall improvement in the machined surface quality.

## Figures and Tables

**Table 1:** Chemical composition of TC18 titanium alloy.

Element	Mo	Cr	Si	N	C	Fe	V	AL
content (Wt%)	4.0~5.5	0.5~1.5	<0.15	<0.0	<0.1	0.5~1.5	4.0~5.5	4.4~5.7

**Table 2:** Mechanical property indicators of TC18 titanium alloy.

Yield Strength (MPa)	Tensile Strength (MPa)	Elongation (%)	Percentage Reduction in Area (%)
1042	1107	14	34.3

**Table 3:** Parameters in the univariate test.

Sample	Cutting Speed/*v*_w_ (m/min)	Feed Rate Per Tool/*f*_z_ (mm/z)	Cutting Depth/*a_p_* (mm)	Amplitude/A (μm)
1–4	20, 30, 40, 50	0.03	0.3	2
5–8	30	0.01, 0.03, 0.05, 0.07	0.3	2
9–12	30	0.03	0.1, 0.2, 0.3, 0.4	2
13–16	30	0.03	0.3	0, 2, 4, 6

**Table 4:** Parameters in the orthogonal test.

Level	Cutting Speed (A)/*v*_w_ (m/min)	Feed Rate Per Tool (B)/*f*_z_ (mm/z)	Cutting Depth (C)/*a_p_* (mm)	Amplitude (D)/A (μm)
1	20	0.01	0.1	0
2	30	0.03	0.2	2
3	40	0.05	0.3	4
4	50	0.07	0.4	6

**Table 5:** Results in the orthogonal test.

No.	Cutting Speed/*v_w_* (m/min)	Feed Rate Per Tooth/*f_z_* (mm/z)	Milling Depth/*a_p_* (mm)	Amplitude/A (μm)	Fx (N)	Fy (N)	Fz (N)	Roughness/Ra (μm)
1	20	0.01	0.1	0	13.3	5.5	33.9	0.157
2	20	0.03	0.2	2	28.3	13.0	49.0	0.390
3	20	0.05	0.3	4	39.7	19.2	53.4	0.650
4	20	0.07	0.4	6	100.0	36.0	122.1	0.594
5	30	0.01	0.2	4	17.7	7.7	33.3	0.189
6	30	0.03	0.1	6	14.3	7.3	29.7	0.201
7	30	0.05	0.4	0	86.1	34.3	104.9	0.223
8	30	0.07	0.3	2	62.9	25.3	82.9	0.401
9	40	0.01	0.3	6	43.3	16.0	65.7	0.340
10	40	0.03	0.4	4	69.5	27.0	84.4	0.322
11	40	0.05	0.1	2	15.0	9.4	30.9	0.202
12	40	0.07	0.2	0	28.5	17.2	40.8	0.258
13	50	0.01	0.4	2	53.4	20.7	70.0	0.288
14	50	0.03	0.3	0	47.8	20.4	62.5	0.268
15	50	0.05	0.2	6	26.3	13.6	39.0	0.239
16	50	0.07	0.1	4	17.1	10.3	33.8	0.293

**Table 6:** Range analysis results of milling force Fx.

	Cutting Speed/*v_w_* (m/min)	Feed Rate Per Tooth/*f_z_* (mm/z)	Milling Depth/*a_p_* (mm/z)	Amplitude/A (μm)
K1	45.330	31.923	14.925	43.933
K2	45.230	39.992	25.173	39.875
K3	39.063	41.748	48.410	35.987
K4	36.152	52.112	77.267	45.981
Range	9.178	20.189	62.342	9.994

**Table 7:** Range analysis results of milling force Fy.

	Cutting Speed/*v_w_* (m/min)	Feed Rate Per Tooth/*f_z_* (mm/z)	Milling Depth/*a_p_* (mm/z)	Amplitude/A (μm)
K1	18.406	12.462	8.133	19.343
K2	18.637	16.915	12.884	17.094
K3	17.432	19.127	20.215	16.043
K4	16.256	22.227	29.500	18.251
Range	2.381	9.765	21.367	3.300

**Table 8:** Range analysis results of milling force Fz.

	Cutting Speed/*v_w_* (m/min)	Feed Rate Per Tooth/*f_z_* (mm/z)	Milling Depth/*a_p_* (mm/z)	Amplitude/A (μm)
K1	64.581	50.722	32.077	60.516
K2	62.713	56.388	40.527	58.198
K3	55.431	57.045	66.101	51.223
K4	51.319	69.889	95.339	64.108
Range	13.262	19.167	63.262	12.885

**Table 9:** Variance analysis results of milling force Fx.

Variance Source	SS	DOF	MS	F Ratio	Significance
*v_w_* (m/min)	252.467	3	84.156	0.824	0.561
*f_z_* (mm/z)	826.576	3	275.525	2.688	0.218
*a_p_* (mm)	9198.972	3	3066.324	30.036	0.010
A (μm)	236.077	3	78.692	0.771	0.582

**Table 10:** Variance analysis results of milling force Fy.

Variance Source	SS	DOF	MS	F Ratio	Significance
*v_w_* (m/min)	14.126	3	4.709	0.991	0.503
*f_z_* (mm/z)	202.314	3	67.438	14.193	0.028
*a_p_* (mm)	1041.100	3	347.033	73.093	0.003
A (μm)	24.460	3	8.153	1.716	0.334

**Table 11:** Variance analysis results of milling force Fz.

Variance Source	SS	DOF	MS	F Ratio	Significance
*v_w_* (m/min)	462.855	3	154.285	0.826	0.561
*f_z_* (mm/z)	787.110	3	262.370	1.405	0.393
*a_p_* (mm)	9744.556	3	3248.185	17.390	0.021
A (μm)	354.230	3	118.077	0.632	0.642

**Table 12:** Variance analysis results of surface roughness.

Variance Source	SS	DOF	MS	F Ratio	Significance
*v_w_* (m/min)	0.097	3	0.032	10.069	0.045
*f_z_* (mm/z)	0.043	3	0.014	1.458	0.125
*a_p_* (mm)	0.096	3	0.032	9.965	0.045
A (μm)	0.044	3	0.015	4.552	0.123

**Table 13:** Surface roughness range analysis results.

	Cutting Speed/*v_w_* (m/min)	Feed Rate Per Tooth/*f_z_* (mm/z)	Milling Depth/*a_p_* (mm/z)	Amplitude/A (μm)
K1	0.448	0.244	0.213	0.227
K2	0.254	0.295	0.269	0.320
K3	0.281	0.329	0.415	0.364
K4	0.272	0.387	0.361	0.343
Range	0.176	0.143	0.201	0.137

## Data Availability

The original contributions presented in this study are included in the article. Further inquiries can be directed to the corresponding author.
